# Daam2 phosphorylation by CK2α negatively regulates Wnt activity during white matter development and injury

**DOI:** 10.1073/pnas.2304112120

**Published:** 2023-08-22

**Authors:** Chih-Yen Wang, Zhongyuan Zuo, Juyeon Jo, Kyoung In Kim, Christine Madamba, Qi Ye, Sung Yun Jung, Hugo J. Bellen, Hyun Kyoung Lee

**Affiliations:** ^a^Department of Pediatrics, Section of Neurology, Baylor College of Medicine, Houston, TX 77030; ^b^Jan and Dan Duncan Neurological Research Institute, Texas Children’s Hospital, Houston, TX 77030; ^c^Department of Biotechnology and Bioindustry Sciences, National Cheng Kung University, Tainan 70101, Taiwan; ^d^Department of Molecular and Human Genetics, Baylor College of Medicine, Houston, TX 77030; ^e^Cancer and Cell Biology Program, Baylor College of Medicine, Houston, TX 77030; ^f^Department of Biochemistry and Molecular Biology, Baylor College of Medicine, Houston, TX 77030; ^g^Department of Neuroscience, Baylor College of Medicine, Houston, TX 77030

**Keywords:** Daam2, CK2α, Wnt, oligodendrocyte, white matter injury

## Abstract

Wnt signaling plays an essential role in developmental and regenerative myelination in the central nervous system. The Wnt signaling pathway is composed of multiple regulatory layers; thus, how these processes are coordinated to orchestrate oligodendrocyte (OL) development remains unclear. Here, we show CK2α, a Wnt/β-catenin signaling Ser/Thr kinase, phosphorylates Daam2, inhibiting its function and Wnt activity during OL development. Intriguingly, we found Daam2 phosphorylation differentially impacts distinct stages of OL development, accelerating early differentiation followed by decelerating maturation and myelination. Application toward white matter injury revealed CK2α-mediated Daam2 phosphorylation plays a protective role for developmental and behavioral recovery after neonatal hypoxia, while promoting myelin repair following adult demyelination. Together, our findings identify a unique regulatory node in the Wnt pathway that regulates OL development via protein phosphorylation-induced signaling complex instability and highlights a new biological mechanism for myelin restoration.

## Significance

Wnt signaling plays a vital role in oligodendrocyte (OL) development and has been implicated as an adverse event for myelin repair after white matter injury. Emerging studies have shed light on multimodal roles of Wnt effectors in the OL lineage, but the underlying molecular mechanisms and modifiable targets in OL remyelination remain unclear. Using genetic mouse development and injury model systems, we delineate a unique stage-specific function of Daam2 in Wnt signaling and OL development via a S704/T705 phosphorylation mechanism and determine a different role of the kinase CK2α in the regulation of OL development and myelin regeneration.

Oligodendrocytes (OLs) are myelin-producing cells that provide functional and metabolic support to axons in the central nervous system (CNS) (1). In white matter disorders, such as hypoxic-ischemic encephalopathy (HIE) and multiple sclerosis (MS), loss of OLs and myelin sheaths are early hallmarks of disease initiation and progression (2, 3). OL precursor cells (OPCs) are recruited to the lesion for tissue repair, but secretory molecules from the adjacent tissue block OL differentiation, thereby limiting remyelination (4–7). Importantly, upregulation of canonical Wnt signaling has been reported in MS patients (8, 9), and aberrant activation of Wnt signaling is well accepted as an adverse event for remyelination. However, manipulating Wnt regulators using genetic models has produced inconsistent outcomes (10, 11), possibly because these Wnt components interact with other pathways, affect transcriptional partners at different stages of OL lineage, or have Wnt-independent functions (7, 10, 12). Therefore, it is imperative to delineate the temporal dynamics and molecular mechanism of Wnt signaling at different stages of OPCs/OLs.

Cytoskeletal remodeling is crucial for stage-specific OL lineage progression. While actin polymerization is required for OL process extension during early differentiation and ensheathment stages, actin depolymerization is necessary for myelin wrapping at the maturation period (13). Formin proteins play a key role in cellular morphogenesis by mediating actin assembly and cytoskeletal remodeling (14). Daam2 [Dishevelled (Dvl)-associated activator of morphogenesis 2] is a formin member that enhances canonical Wnt signaling during embryonic spinal cord patterning (15). Daam2 overexpression greatly inhibits OL differentiation, where loss of Daam2 promotes differentiation during development and after white matter injury (16, 17). Consistent with this finding, Daam2 is up-regulated in demyelinated lesions in conjunction with a higher Wnt tone in HIE and MS patients (11, 16, 17). However, we recently found that loss of Daam2 leads to an abnormal myelin formation which returns to normal at an older stage (18). Hence, these studies raise two important questions. 1) Does Wnt signaling play a negative role in early differentiation and a positive role during maturation and myelination? 2) What are the molecular mechanisms that regulate the activity of Daam2?

CK2, a serine/threonine kinase, interacts with multiple Wnt components and positively regulates Wnt activity (19–21). While it is established that CK2 subunits are essential for brain development and OPC production (22, 23), their potential roles and targets in OL differentiation via Wnt signaling remain to be determined. In this study, we discovered that phosphorylated Daam2 at S704/T705 attenuates Wnt/β-catenin signaling in OL lineage and promotes OL differentiation but subsequently decelerates myelination. We identified CK2α as the kinase that phosphorylates Daam2 followed by weakening Wnt signaling complex. Moreover, in white matter injury models, both CK2α overexpression and Daam2 phosphorylation were found to promote tissue repair. Our findings establish a unique role for CK2α in blunting the adverse effect of Daam2-mediated Wnt signaling for OL differentiation, suggesting a new regulatory pathway for white matter diseases.

## Results

### Daam2 Phospho-Mimetic Mutant Promotes OL Differentiation.

**Fig. 1.**
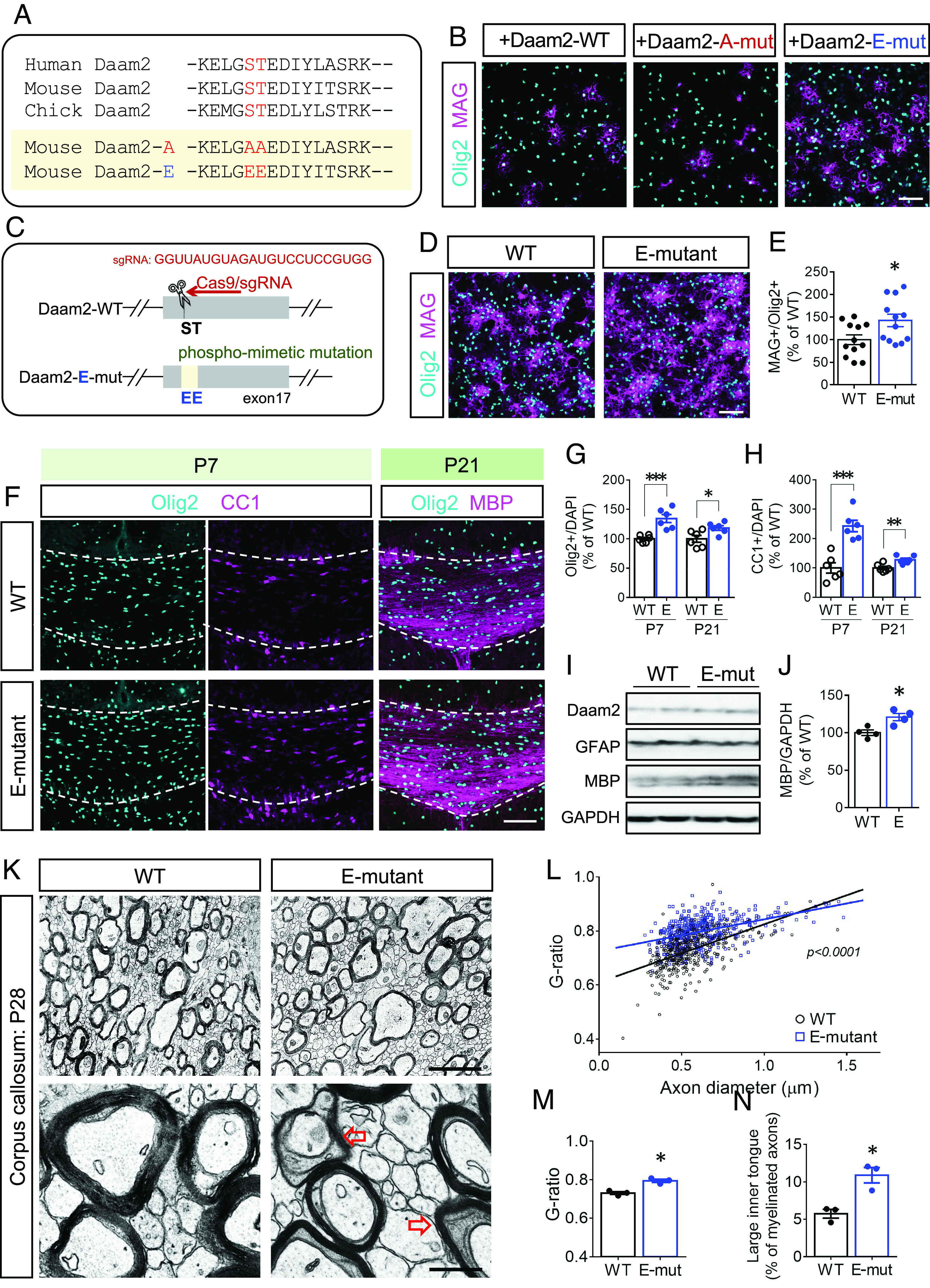
Phospho-mimetic mutation of Daam2 accelerates OL differentiation but delays myelination. (*A*) Phosphorylation motif of Daam2 in human, mouse and chick are shown. For following experiments, phospho-mimetic (E-mut) and phospho-null mutation (A-mut) of Daam2 were used. (*B*) Daam2-WT, Daam2-E-mut, and Daam2-A-mut were transfected into primary OPCs followed by differentiation for 2 d. (*C*) E-mutant mice were produced with CRISPR-Cas9-based technique. The guide RNA sequence for the E-mutation is shown. (*D*) In vitro OL differentiation was assessed using WT and the E-mutant OL culture. (*E*) The number of MAG^+^ cells for 2-d differentiation was quantified. (*F*–*H*) P7 and P21 brains from WT and the E-mutant mice were analyzed by immunofluorescence staining. Olig2^+^ and CC1^+^ (APC^+^) cells in the corpus callosum were counted. (*I* and *J*) P21 cortical tissues containing the corpus callosum from WT and the E-mutant were analyzed by western blot. The MBP protein levels were quantified. (*K*) The myelin structure in the corpus callosum from WT and the E-mutant mice at P28 were subjected to electron microscopy. (*L* and *M*) Axon diameter and g-ratio from each myelinated axon were measured. (*N*) Axons with enlarged inner tongue microstructure (red arrows in *K*) were also counted. The data were collected from at least 3 independent experiments or animals for each group. Data were presented as mean ± SEM and normalized to WT. *P < 0.05, **P < 0.01, ***P < 0.001 vs. WT. [Scale bar, 100 μm in *B*, *D*, and *F*; 2 μm (*Upper*) and 0.5 μm (*Lower*) in *K*.]

To identify possible regulators of Daam2, we performed Daam2 immunoprecipitations (IP) on mouse cortical tissues followed by mass spectrometric (IP-MS) analysis. We found that residues S704 and T705 in the FH2 domain of Daam2 are phosphorylated. The amino acid sequence is highly conserved among species in Daam2 orthologs (Fig. 1*A*). The S704 phosphorylation (S656 in humans) of Daam2 has been previously reported (24, 25). To investigate the effect of phosphorylation, we introduced phospho-null mutant (S704A/T705A, denoted as A-mutant), or phospho-mimetic mutant (S704E/T705E, denoted as E-mutant) into Daam2, and transfected them into primary OPCs (Fig. 1 *A* and *B*). We confirmed that wild-type (WT) Daam2 suppresses OL differentiation as evidenced by a reduced number of mature OL markers including MAG^+^ and MBP^+^ cells after 2 and 4 d of OL differentiation, respectively (*SI Appendix,* Fig. S1 *A*–*C*). The overexpression of the A-mutant had a comparable negative effect on OL differentiation, whereas the E-mutant increased the number of MAG^+^ and MBP^+^ cells with morphological complexity (Fig. 1*B* and *SI Appendix,* Fig. S1 *D*–*F*).

To examine the endogenous function of Daam2 phosphorylation in vivo, we generated knock-in mice bearing the Daam2 E-mutation in the endogenous locus (Fig. 1*C*). In accordance with our in vitro findings, OPCs from the E-mutant brain produced more MAG^+^ and MBP^+^ OLs than WT brains (Fig. 1 *D* and *E* and *SI Appendix,* Fig. S1*G*). During the myelinogenesis period in the brain, we also found more Olig2^+^ OL lineage cells and CC1^+^ (APC^+^) mature OLs in the E-mutant corpus callosum at P7 and P21 than in the WT (Fig. 1 *F*–*H* and *SI Appendix,* Fig. S1*J*). In vitro data indicate that E-mutation did not affect OPC proliferation (*SI Appendix,* Fig. S1*H*) but facilitates OPC specification and OL lineage progression from neural stem cells (NSCs; *SI Appendix,* Fig. S1*I*). In addition, MBP levels were up-regulated in the E-mutant brain (Fig. 1 *I* and *J* and *SI Appendix,* Fig. S1*K*), yet the thickness of the myelin sheath in the corpus callosum was reduced (Fig. 1 *K*–*M*). We also observed more axons surrounded by enlarged inner tongue structures, the cytoplasmic space between the axons and the myelin, in the E-mutant corpus callosum at P28 (Fig. 1 *K* and *N*; arrows in Fig. 1*K*). However, myelin integrity in the E-mutant was restored in adulthood (*SI Appendix,* Fig. S1*L*), suggesting that it takes a longer time to complete axon ensheathment in the E-mutant brain. Yet, we did not observe significant differences between the E-mutant and WT brains with respect to astrocytes, another group of Daam2-expressing cells in the CNS (*SI Appendix,* Fig. S1 *M*–*O*). Our results indicate that the Daam2 S704/T705 phosphorylation could alter Daam2 functions in the OL lineage progression.

### Dynamic Transcriptomic Remodeling in Early and Late OLs by Daam2 Phosphorylation.

**Fig. 2.**
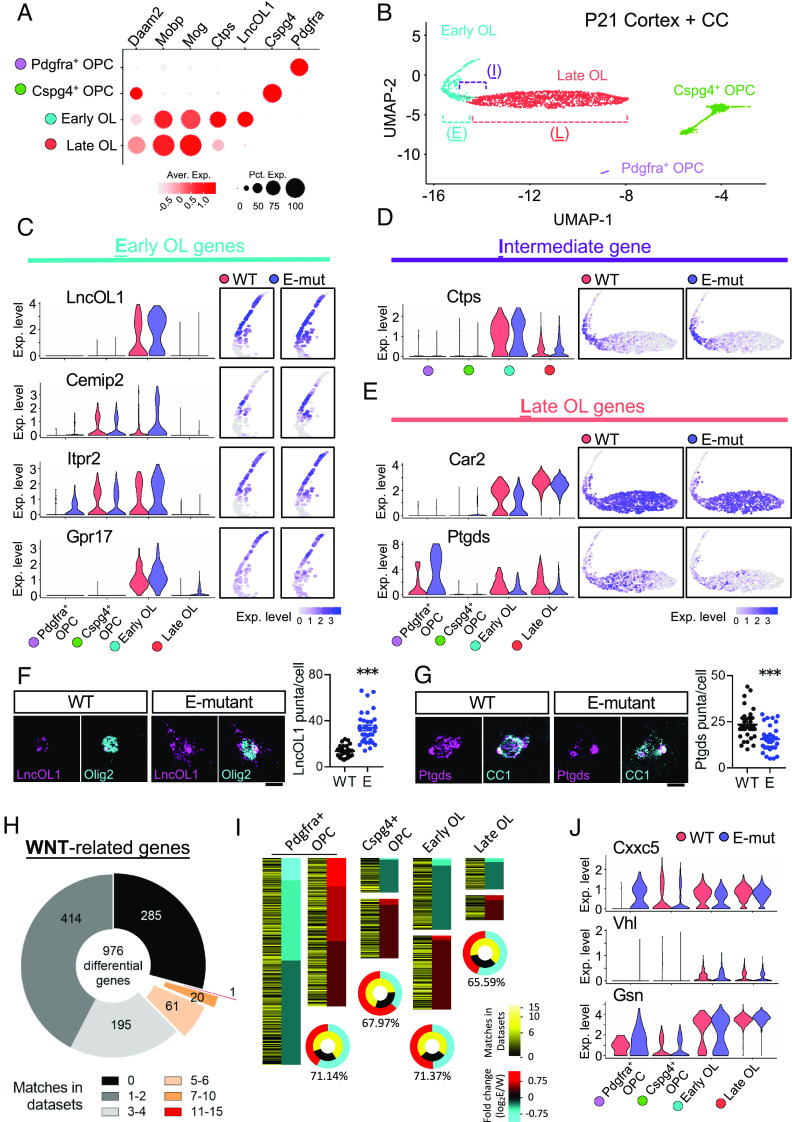
Early genes are upregulated but late genes are downregulated in the E-mutant OLs by scRNA-seq analysis. (*A*) The cell identities of clusters from scRNA-seq using in P21 brains were determined by specific markers, *Pdgfra* (OPCs), *Cspg4* (OPCs), *LncOL1*, *Ctps* (early OLs), *Mog*, and *Mobp* (late OLs). (*B*) OPC/OL clusters were visualized by dimension reduction plot, Uniform Manifold Approximation and Projection (UMAP). (*C*–*E*) The locations of cells expressing the genes were displayed in UMAP of WT and the E-mutant. Early OL genes (*LncOL1*, *Cemip2Itpr2*, and *Gpr17,* *Left*), intermediate gene (*Ctps*), late/maturation genes (*Car2*, and *Ptgds*) of WT and the E-mutant are shown by violin plots (*Right*; also see Dataset S1). (*F* and *G*) *LncOL1 and Ptgds* RNA levels in OLs of P21 mice were validated by RNAscope. (*H*) 976 differentially expressed genes (DEGs; Log2-Fold change > 0.25 and *P* value < 0.05) between WT and the E-mutant were aligned with 28 datasets containing Wnt-related gene list (Dataset S2). The number of genes that matched with Wnt datasets are shown in different divisions in the pie chart. (*I*) For 4 clusters, genes upregulated in E-mutant are marked in red, and downregulated in cyan. Genes that match Wnt datasets are labeled in dark to bright yellow (low to high matches). (*J*) Wnt-related genes, Cxxc5 (Wnt inhibitor gene), Vhl and Gsn are shown in violin plots.

To identify the molecular signatures that underlie the observed prodifferentiation effects of the Daam2 E-mutant, we performed transcriptome analysis using 10× Genomics single-cell RNA sequencing (scRNA-seq) in P21 brains (GEO accession: GSE236976) (26). After unbiased clustering, four clusters of OL lineage cells (*Pdgfra*^+^ OPCs, *Cspg4*^+^ OPCs, early differentiated OLs, and late differentiated OLs) were identified using specific OL stage markers such as *Pdgfra*, *Cspg4*, *LncOL1,* and *Mobp* (Fig. 2 *A* and *B*) from WT and E-mutant brains. Specifically, early and late gene signatures for OL progression were identified. For the early genes (those correlated with the early OL marker *LncOL1*), *Cemip2, Itpr2, and Gpr17* were significantly up-regulated by the E-mutation in early OLs compared to WT (Fig. 2*C* and Dataset S1). In contrast, the late genes (as mature OL markers), *Car2*, *Ptgds*, *Grm3*, and *Aspa* were significantly down-regulated by the E-mutation in both early and late OL clusters (Fig. 2*E* and Dataset S1). The RNA expression of *LncOL1* and *Ptgds* in the OL lineage was also validated (Fig. 2 *F* and *G*). Notably, the intermediate gene *Ctps* demonstrated no significant difference (Fig. 2*D* and Dataset S1), suggesting a transition in OL lineage progression in the E-mutant. Collectively, these transcriptomic changes are consistent with our observation that OL differentiation and myelination are affected in the E-mutant (Fig. 1).

To explore how Daam2 phosphorylation regulates OL lineage progression, differentially expressed genes (DEGs) between WT and the E-mutant were subjected to gene ontology (GO) and Kyoto Encyclopedia of Genes and Genomes (KEGG) enrichment analysis. DEGs downregulated in the E-mutant OPCs/OLs are enriched for ER-mediated protein processing and lipid/cholesterol metabolism (*SI Appendix,* Fig. S2). In contrast, DEGs up-regulated by the E-mutation are involved in multiple signaling processes, including the Wnt pathway. Since Daam2 is a positive modulator of canonical Wnt signaling, we further examined whether these DEGs result from the alteration of Wnt signaling. Crosschecking with 28 published datasets containing Wnt-related genes (Dataset S2) revealed that over 70% of the DEGs are associated with Wnt signaling (Fig. 2*H*). We also observed higher portions of Wnt-related DEGs in *Pdgfra*^+^ OPCs and early OLs than in late OLs (Fig. 2*I*). For example, *Cxxc5*, a Wnt negative feedback regulator and a transcriptional activator for myelin genes, was elevated in *Pdgfra*^+^ OPCs but reduced in early OLs in the E-mutant OLs, suggesting a temporally specific regulatory mechanism of Wnt signaling during OL differentiation (Fig. 2*J* and Dataset S1). Moreover, we also confirmed dynamic regulation of *Vhl* and *Gsn* expression from Wnt-regulated DEGs in the E-mutant OLs (Fig. 2*J* and Dataset S1), which intriguingly were previously identified as critical for promoting OL differentiation mediated by Daam2 (17, 18). Together, these results suggest that phosphorylation of Daam2 regulates Wnt signaling in the entire OL lineage and controls OL differentiation and maturation respectively.

### Daam2 Regulates Wnt Signaling in the OL Lineage in a Stage-Specific Manner.

**Fig. 3.**
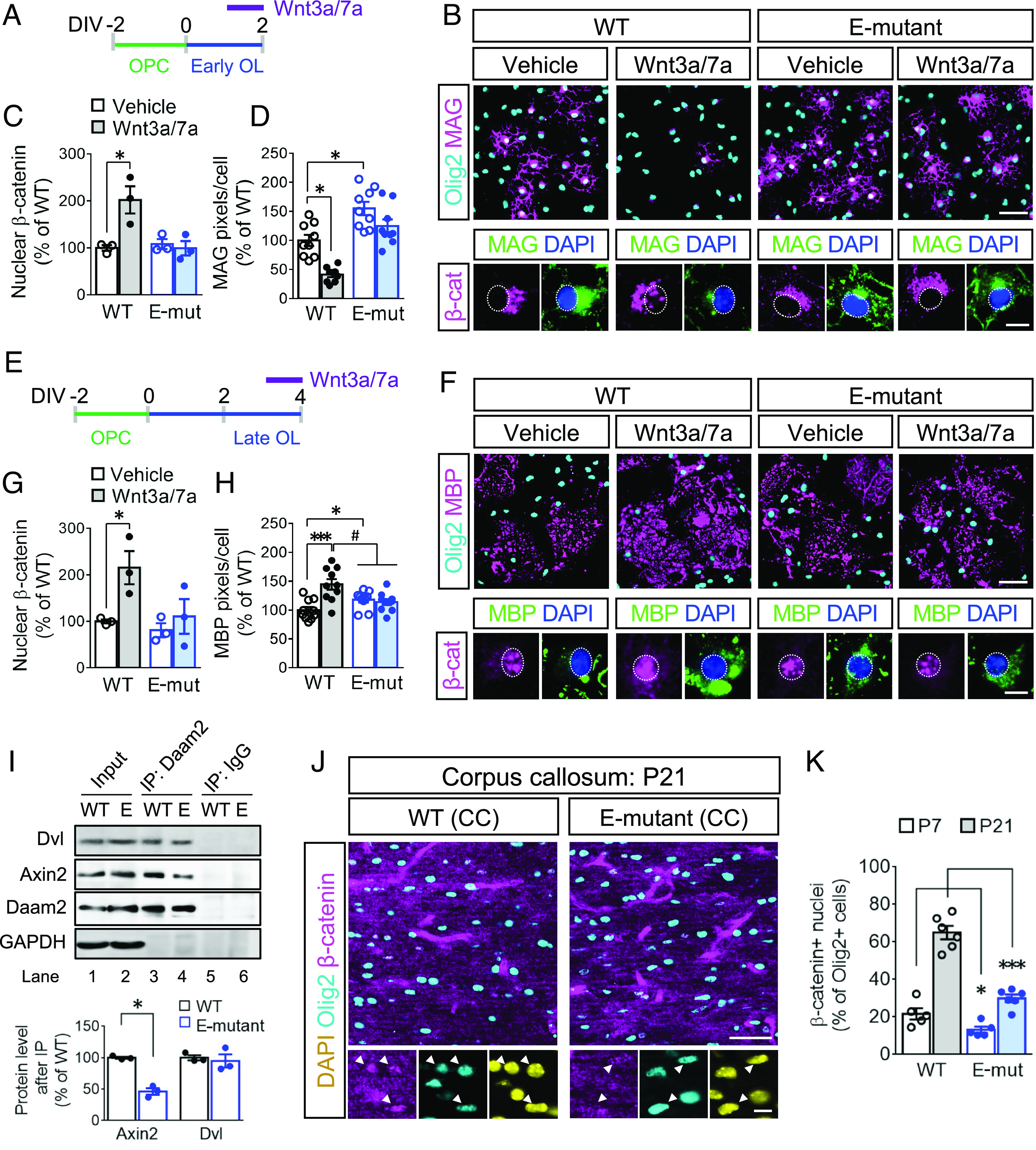
Exogenous Wnt/β-catenin signaling is blocked in the E-mutant OLs. (*A*) Wnt3a/7a were treated to early OLs of WT and the E-mutant for 24 h prior to harvesting. (*B*) In vitro differentiation by MAG expression in early OLs after Wnt3a/7a treatment were assessed by immunofluorescence staining. The subcellular locations of β-catenin were also visualized. (*C*) β-catenin levels in the nucleus fractions of early OLs were analyzed and quantified by western blot (*SI Appendix,* Fig. S3*A*). (*D*) The MAG expression in B was quantified. (*E*–*H*) Similar to A-D, Wnt3a/7a were treated to late OLs of WT and the E-mutant. The nuclear β-catenin (*G*) and MBP expression (*H*) in late OLs were measured (*SI Appendix,* Fig. S3*B*). (*I*) Daam2 IP was conducted using WT and the E-mutant brain at P21, Wnt complex components, Dvl and Axin2 were blotted and the protein enrichment was quantified in the lower panel. (*J* and *K*) β-catenin distributions were evaluated by immunofluorescence staining in the WT and E-mutant corpus callosum at P7 and P21. Nuclear β-catenin was identified by overlapping with DAPI^+^ nuclei and by showing higher intensity than the surroundings. OL lineage cells were labeled by Olig2. The data were collected from at least 3 independent experiments or animals for each group. Data were presented as mean ± SEM and normalized to WT/vehicle. *P < 0.05, ***P < 0.001 vs. WT/vehicle; ^#^*P* < 0.05, vs. WT/Wnt. [Scale bar, 50 μm (*Upper*), 10 μm (*Lower*) in *B*, *F*, and *J*.]

We next investigated whether Daam2 phosphorylation regulates Wnt activity in the OL lineage in a stage-specific manner. To determine endogenous Wnt activity in primary OLs, we assessed the nuclear β-catenin level at differentiation day 2 (early; Fig. 3*A*) and day 4 (late; Fig. 3*E*). Interestingly, early OLs showed a large amount of cytosolic β-catenin (*SI Appendix,* Fig. S3*A*), while more β-catenin accumulated in the nucleus in late OLs (*SI Appendix,* Fig. S3*B*). To further induce canonical Wnt signaling, we treated early and late OLs with canonical Wnt ligands, Wnt3a and Wnt7a, which caused a two-fold increase in nuclear β-catenin levels (Fig. 3 *B*, *C*, *F*, and *G* and *SI Appendix*, Fig. S3 *A* and *B*, lane 5 vs. 6). In contrast, nuclear β-catenin levels remained unchanged in E-mutant OLs after Wnt3a/7a treatment, indicating that Daam2 phosphorylation severely attenuated ligand-based Wnt/β-catenin signaling (*SI Appendix,* Fig. S3 *A* and *B*, lane 7 vs. 8). To further understand the relationship between Wnt activity and OL differentiation, we examined early differentiation vs. late/terminal differentiation after Wnt3a/7a treatment. Wnt3a/7a greatly reduced early differentiation, with less MAG expression in the WT than in the E-mutant (Fig. 3 *B*–*D*). Wnt3a/7a treatment also increased MBP levels and enlarged membrane structures at a later stage of OL differentiation in the WT (Fig. 3 *F*–*H* and *SI Appendix,* Fig. S3*B*, lane 1 vs. 2), while E-mutant OLs were insensitive to Wnt ligands treatment (*SI Appendix,* Fig. S3*B*, lane 3 vs. 4).

Given that Daam2 is required for Wnt signalosome complex formation, we investigated whether Daam2 phosphorylation interferes its interaction with Wnt components. Although the interaction of Daam2 and Dvl was not altered by the E-mutation in the brain, there was a reduction in the association between Axin2 and the Daam2 E-mutant (Fig. 3*I*). This evidence explains that Daam2–Axin2 interaction is important for Daam2 phosphorylation-mediated Wnt signaling cascade. To validate our in vitro findings, we assessed the β-catenin protein expression pattern in WT and the E-mutant corpus callosum at P7 and P21 (Fig. 3*J*). At P7, nuclear β-catenin was observed in ~20% of Olig2^+^ population of cells in WT which increased to ~70% at P21 (Fig. 3 *J* and *K*). In comparison, the overall levels of nuclear β-catenin were down-regulated in the E-mutant mice. On the other hand, Tcf7l2 (also known as Tcf4), an important cotranscription factor with β-catenin in OLs (11), is up-regulated in the E-mutant (*SI Appendix,* Fig. S3 *C*–*F*). The inverse correlation between the expression of nuclear β-catenin and Tcf7l2 in the E-mutant and WT suggests dynamic regulation of Wnt signaling in OL lineage progression. Together, these observations demonstrate dynamic changes in the machinery and function of Wnt/β-catenin signaling in early vs. late OLs, and this pathway is affected by Daam2 phosphorylation.

### CK2α-Mediated Daam2 Phosphorylation Stimulates OL Differentiation.

**Fig. 4.**
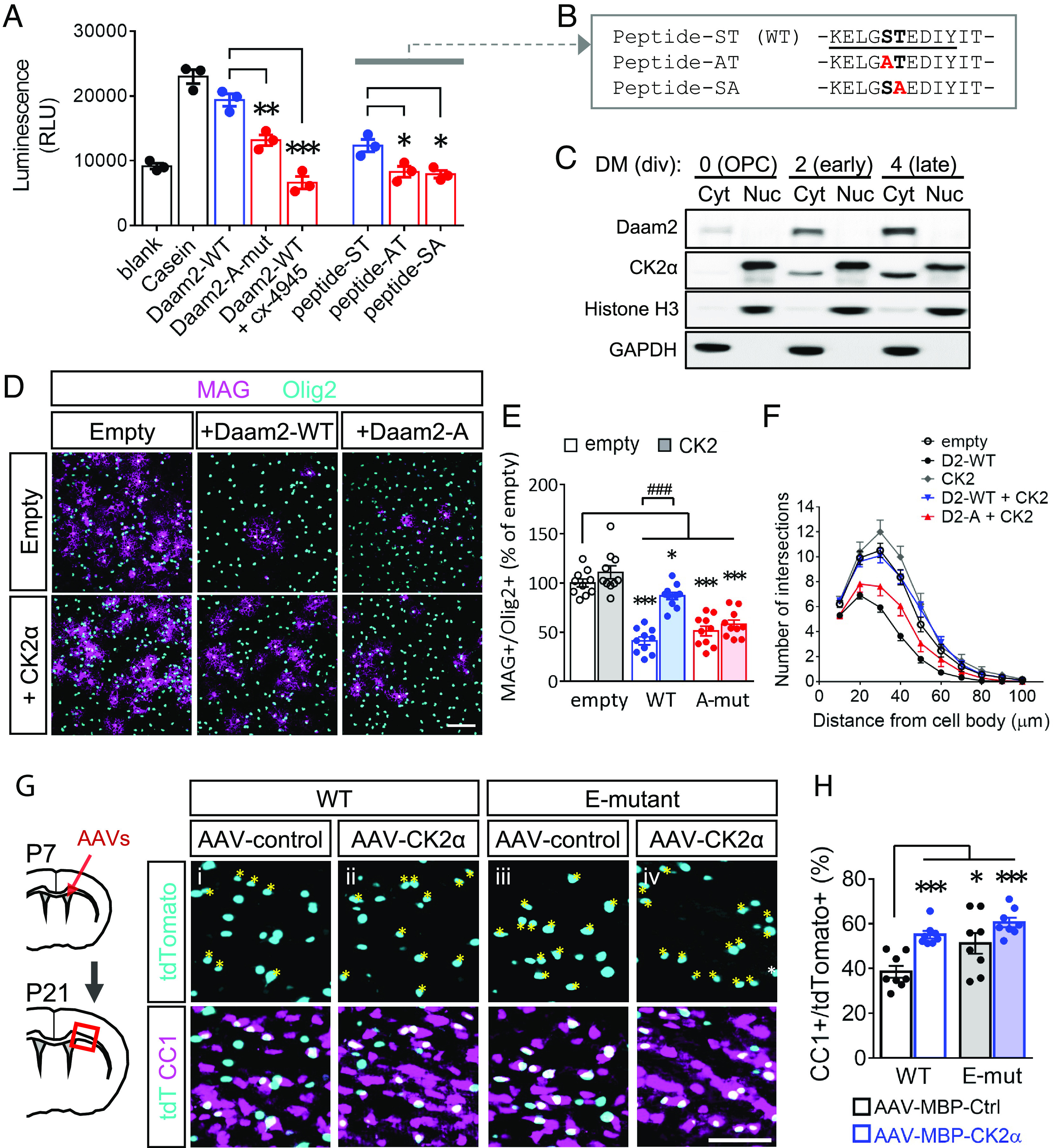
CK2α phosphorylates Daam2 and facilitates OL differentiation. (*A* and *B*) Purified Daam2 proteins and synthesized peptides (sequences shown in *B*) were subjected to in vitro CK2 kinase assay. Blank: no substrate; Casein: positive control. (*C*) Endogenous CK2α expression was blotted in nucleus and cytosol fractions from different stages of OPC/OL culture. (*D*) In vitro differentiation was evaluated by immunofluorescence staining after transducing Daam2 and CK2α. (*E*) The number of MAG^+^/Olig2^+^ cells were counted. (*F*) The number of processes extended from a cell body at different distances was analyzed by Sholl analysis. (*G* and *H*) AAV–MBP–CK2α and AAV–MBP–control were injected intracerebroventricularly into P7 pups respectively. CC1^+^ cells with tdTomato reporter were visualized by immunofluorescence staining. % of injected cells that became mature OLs was quantified as CC1^+^/tdTomato^+^. The CC1^+^/tdTomato^+^ cells are marked with asterisks. Data from at least 3 independent experiments or animals for each group were presented as mean ± SEM and normalized to empty or WT/AAV–control. *P < 0.05, **P < 0.01, ***P < 0.001 vs. WT proteins in *A*, vs. empty in *E*, vs. WT/AAV–control in *H*; ^###^*P* < 0.001 vs. Daam2-WT. (Scale bar, 100 μm in *D*; 50 μm in *G*.)

To identify the kinases responsible for S704/T705 phosphorylation, we conducted motif analysis by using the NetPhos3.1 database (27). We found CK2 among the candidates from IP-MS data (*SI Appendix,* Fig. S4 *A* and *B*), and we confirmed that its major catalytic subunit, CK2α, interacts with Daam2 in primary OLs (*SI Appendix,* Fig. S4*C*). To investigate whether CK2α directly phosphorylates Daam2, we performed in vitro kinase assays using either purified Daam2 proteins or synthesized peptides containing the phosphorylation site (Fig. 4*A*). CK2α phosphorylates both Daam2 and the synthesized peptides, and both the S704A or T705A mutation blocked this phosphorylation (Fig. 4 *A* and *B*). We further examined whether CK2α phosphorylates Daam2 in primary OLs and found that CK2α overexpression increased phospho-serine levels of WT–Daam2, but not of the A-mutant protein levels (*SI Appendix,* Fig. S4*D*). Moreover, CK2α and Daam2 proteins were sequentially up-regulated in the cytosolic compartment concomitant with OL lineage progression (Fig. 4*C*). These results suggest a regulatory pathway of CK2α–Daam2 in OLs.

Next, we tested whether CK2α facilitates OL differentiation via Daam2 phosphorylation. Although CK2α overexpression showed an increasing trend in OL differentiation in vitro, CK2α reversed the inhibitory effect of Daam2 (Fig. 4 *D*–*F*). In agreement with previous data, OL differentiation was also reduced by CK2 inhibitors, cx-4945 and DMAT (*SI Appendix,* Fig. S5 *A* and *B*) or a CK2α dominant negative mutant (K68A; dnCK2α; *SI Appendix,* Fig. S5 *C* and *D*), indicating a requisite role of CK2 kinase function. To examine the function of CK2α in mice in vivo, we created adeno-associated viruses (AAVs) carrying the CK2α gene as well as the tdTomato reporter under the control of the MBP promoter to express the gene selectively in differentiating OLs. The virus was injected intracerebroventricularly into mouse brains at P7 (*SI Appendix,* Fig. S5*E*). The upregulation of CK2α by AAV-CK2α was confirmed in tdTomato^+^ cells when compared to the AAV–controls using the WT brain (*SI Appendix,* Fig. S5*F*). Additionally, most of the tdTomato^+^ cells were restricted to the corpus callosum at P21 as Olig2^+^ OL lineage (*SI Appendix,* Fig. S5 *G* and *H*). As a result, OL-specific overexpression of CK2α increased the abundance of CC1^+^ cells among tdTomato^+^ cells (Fig. 4 *G* and *H*, *i* vs. *ii*), confirming the stimulatory role of CK2α in OL differentiation in vivo.

To further validate the CK2α–Daam2 pathway in vivo, we also introduced the AAVs into the E-mutant brains. As the E-mutant mice displayed early OL differentiation, there were more CC1^+^/tdTomato^+^ cells in the E-mutant brains receiving the AAV–control as compared to the WT brains (Fig. 4 *G* and *H*, *i* vs. *iii*). However, the E-mutant brains receiving AAV–CK2α did not show further enhancement by CK2α overexpression and by the E-mutation, respectively (Fig. 4 *G* and *H*, *iv* vs. *ii* and *iii*), confirming that CK2α and Daam2 phosphorylation share the same pathway in this model. Therefore, these data provide compelling evidence that CK2α promotes OL differentiation by phosphorylating Daam2.

### Daam2 Phosphorylation Improves Functional Recovery after Neonatal Hypoxic Injury.

**Fig. 5.**
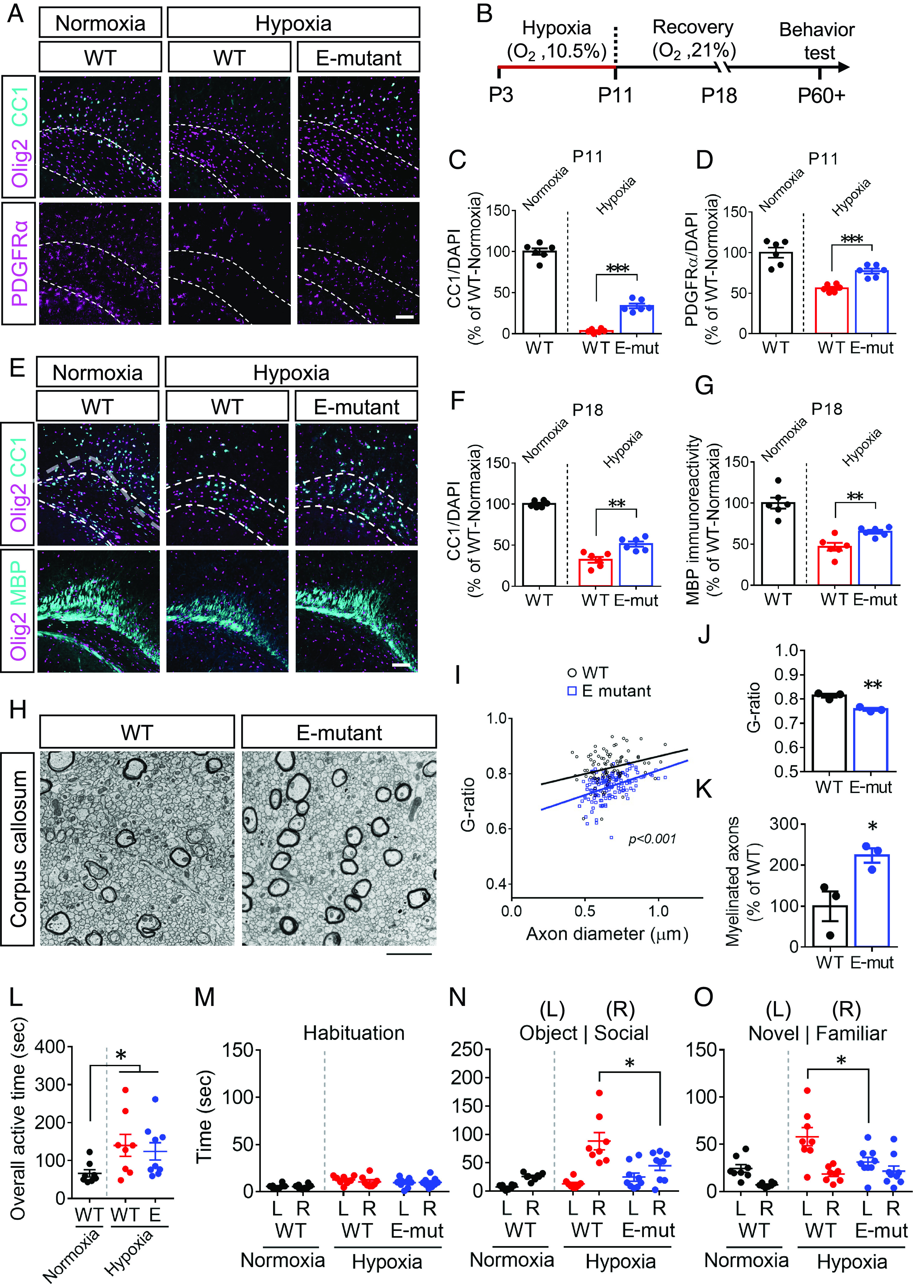
The E-mutant mice show better functional recovery after postnatal hypoxic injury. The brains of P11 (*A*–*D*) and P18 (*E*–*G*) WT and the E-mutant mice, that suffered from postnatal hypoxic injury (from P3 to P11), were analyzed by immunofluorescence staining. The numbers of Olig2^+^, CC1^+^ and PDGFRα^+^ cells were counted within the corpus callosum (dash line), and the immunoreactivity of MBP was measured. (*H*) The myelin structure in the corpus callosum from WT and the E-mutant mice at P18 after postnatal hypoxic injury were subjected to electron microscopy. (*I* and *J*) Axon diameter and g-ratio from each myelinated axon were measured. (*K*) The number of myelinated axons was counted. (*L*–*O*) The social behaviors of 2-mo-old mice were evaluated by 3-chamber sociability test, including overall active time during tests (*L*), habituation (*M*), preference to an object or a mouse (*N*), and preference for a new stranger mouse or a familiar one (*O*). Data from at least 3 (*H*–*K*) or 6 animals for each group were presented as mean ± SEM. *P < 0.05, **P < 0.01, ***P < 0.001. (Scale bar, 100 μm in *A* and *E*; 2 μm in *H*.)

Given that Wnt signaling is up-regulated in white matter lesions of human HIE (16), we next evaluated whether attenuating Daam2 function by modulating its phosphorylation state improves recovery in white matter injury models. In a mouse model of neonatal injury that mimics human HIE (16, 28), brain development was impaired in a hypoxic environment (10.5% O_2_) from P3 to P11 (Fig. 5 *A* and *B*). OL differentiation was significantly impaired as CC1^+^ OLs were barely detected in the corpus callosum immediately after hypoxic injury at P11 (Fig. 5*C*) in conjunction with Daam2 upregulation (*SI Appendix,* Fig. S6*A*). Notably, hypoxia also reduced the number of PDGFRα^+^ OPCs (Fig. 5*D*). In comparison with Daam2 WT controls, there were more CC1^+^ mature OLs and PDGFRα^+^ OPCs in the E-mutant corpus callosum after injury (Fig. 5 *C* and *D*). Moreover, after a 7-d recovery period postinjury, more CC1^+^ OLs were generated in the E-mutant brain compared to WT (Fig. 5 *E* and *F*). MBP immunoreactivity at the cingulum, an early myelination event, was also higher in the E-mutant mice than in WT (Fig. 5*G*). Moreover, we observed significantly more myelinated axons and slightly increased myelin sheath thickness in the E-mutant corpus callosum compared to WT (Fig. 5 *H*–*K*). There was no difference in the development of neurons and astrocytes between WT and E-mutant brains after injury at P18 (*SI Appendix,* Fig. S6 *C* and *D*).

White matter development and neural connectivity are closely related to social behaviors (29). Both human HIE patients and rodents with neonatal hypoxia display functional deficits in childhood and show long-term effects in adolescence and adulthood, such as ADHD-like behaviors and social problem (28, 30, 31). To investigate whether Daam2 phosphorylation is critical for social behavior development after neonatal hypoxic injury, mice were challenged under 10.5% O_2_ hypoxic condition from P3 to P11 and then subjected to a three-chamber sociability test at 2 mo of age (Fig. 5*B*). Similar to previous findings, mice with neonatal hypoxic injury were more active than normal mice (Fig. 5*L*), despite that there is no behavioral difference between WT and E-mutant mice during habituation (Fig. 5*M*). Neonatal hypoxia WT mice showed a higher preference for a stranger mouse over a novel object (Fig. 5*N*), and spent more time with a stranger mouse than with a familiar one (Fig. 5*O*). In contrast, the Daam2 E-mutation mitigated abnormal social preferences induced by hypoxia injury (Fig. 5 *N* and *O*). These data suggest a beneficial role of Daam2 phosphorylation for developmental and behavioral functional recovery after neonatal hypoxic injury.

### CK2α-Induced Daam2 Phosphorylation Facilitates Myelin Repair after Lysolecithin-Induced Demyelination.

**Fig. 6.**
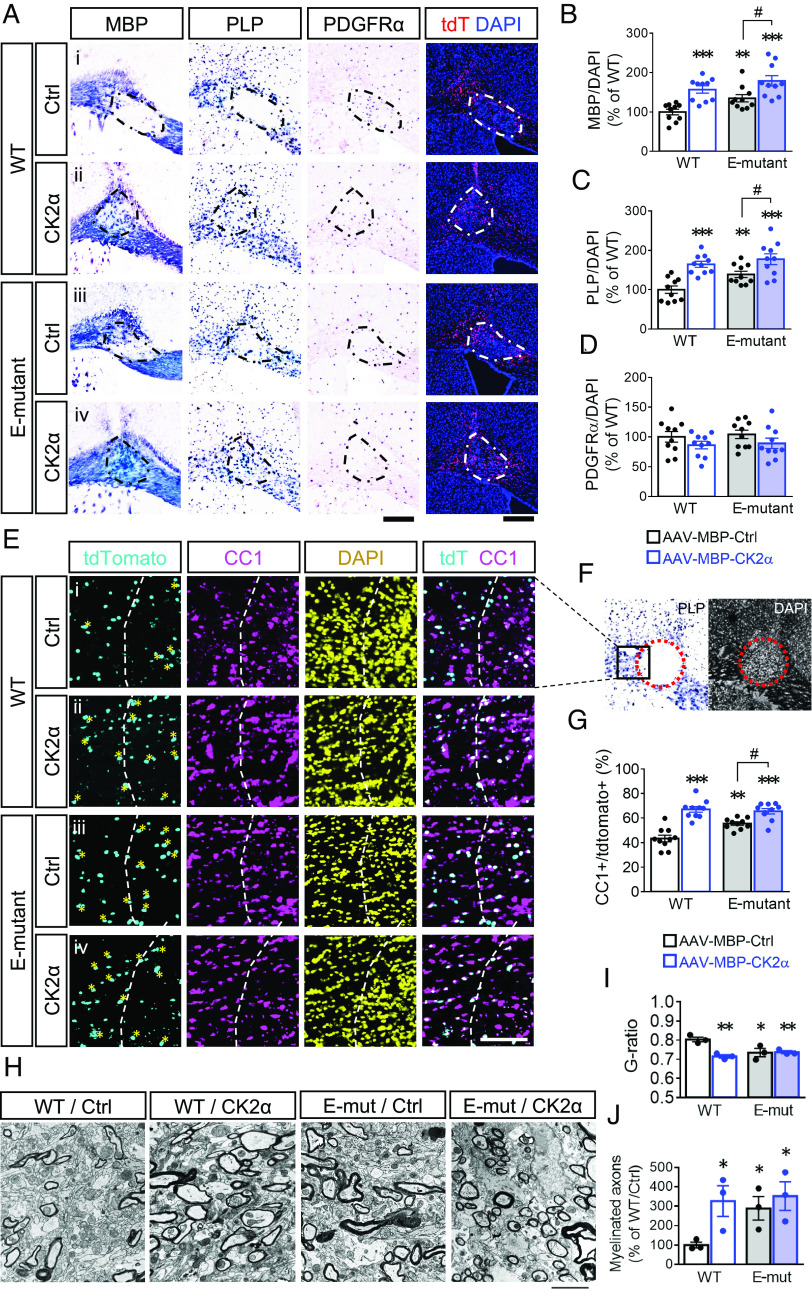
The E-mutant mice show improved tissue repair after lysolecithin-induced demyelination. At 14 d after injecting lysolecithin and AAVs into corpus callosum in adult WT and E-mutant mice, brain tissues containing the lesions were assessed by in situ hybridization for *MbpPlp* and *Pdgfrα* (*A*–*D*). The lesions were visualized by tdTomato reporter and accumulative DAPI. The number of *Mbp*^+^*Plp*^+^*Pdgfrα*^+^ cells, and DAPI in the lesion (dash line) area were counted. (*E*–*G*) The lesioned areas were also analyzed by immunofluorescence staining for CC1^+^ and tdTomato^+^ infected cells. % of injected cells that became mature OLs was quantified as CC1^+^/tdTomato^+^. The CC1^+^/tdTomato^+^ cells are marked with asterisks. (*H*) The myelin structure in the corpus callosum from WT and E-mutant mice at 14 d after receiving lysolecithin and AAVs were subjected to electron microscopy. (*I*) G-ratio from each myelinated axon was measured. (*J*) The number of myelinated axons was counted. Data from at least 10 animals for each group were presented as mean ± SEM. *P < 0.05, **P < 0.01, ***P < 0.001 vs. WT/AAV–control, ^#^*P* < 0.05 vs. E-mut/AAV–control. (Scale bar, 200 μm in *A*; 100 μm in *E*; 2 μm in *H*.)

We previously discovered that Daam2 is expressed in OPCs in human MS lesions (17). To examine the function of Daam2 phosphorylation via CK2α in a mouse model of demyelination that mimics MS (32), we injected 2% lysolecithin with AAV–MBP–CK2α and –control virus in the corpus callosum of E-mutant and WT brains, respectively (Fig. 6*A*). After 14 days postinjury (dpi), we confirmed upregulation of Daam2 (*SI Appendix,* Fig. S6*B*) and evaluated remyelination surrounding the lesion. OL-specific CK2α overexpression significantly increased the number of *Mbp*^+^ and *Plp*^+^ cells around the lesions compared to the control (Fig. 6 *A*–*C*, *i* vs. *ii*), with no difference in *Pdgfra*^+^ cell numbers. Specifically, there were more CC1^+^ OLs among tdTomato^+^ cells infected with AAV–MBP–CK2α than those with AAV–control (Fig. 6 *E*–*G*, *i* vs. *ii*). On the other hand, there were more *Mbp*^+^ and *Plp*^+^ OLs for tissue repair in the E-mutant brains compared to the WT brains (Fig. 6 *A*–*C*, *i* vs. *iii*). The tdTomato^+^ cells infected with AAV–control generated a higher portion of CC1^+^ OLs in the E-mutant brains than in the WT brains (Fig. 6 *E*–*G*, *i* vs. *iii*). Similar to our observations in developing brains (Fig. 4 *G* and *H*), CK2α overexpression in the E-mutant OLs did not show additional or synergistic effect for white matter repair as compared to that in WT OLs in the lesions (Fig. 6 *E*–*G*, *ii* vs. *iv*). Moreover, we observed more myelinated axons as well as thicker myelin sheaths in the corpus callosum with CK2α overexpression and/or E-mutation (Fig. 6 *H*–*J*). There is no difference in reactive astrocytes between E-mutant and WT brains at 14 dpi (*SI Appendix,* Fig. S6 *E*–*G*). These findings demonstrate that the CK2α–Daam2 pathway facilitates remyelination after white matter injury.

## Discussion

**Fig. 7.**
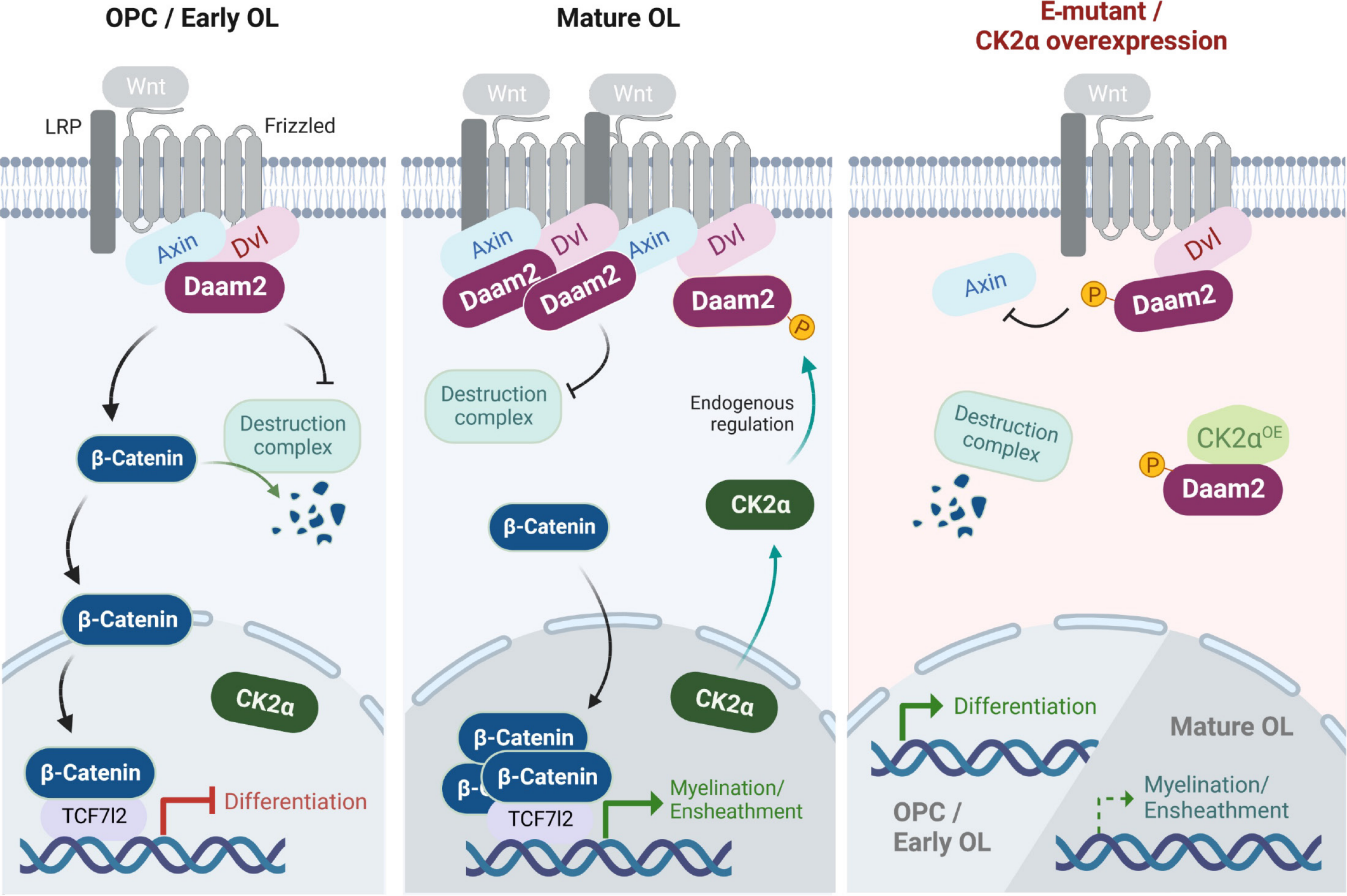
The molecular regulation of Wnt signaling in the OL lineage by Daam2 phosphorylation. The activity of Daam2-mediated Wnt signaling is low in OPCs and early OLs (*Left*) but upregulated in mature OLs (*Middle*). When ligands trigger Wnt signaling, the destruction complex is inhibited, allowing β-catenin to enter the nucleus where it inhibits or enhances the expression of differentiation and myelination/ensheathment genes, respectively, in conjunction with Tcf7l2. In early OLs, CK2α remains in the nucleus and does not interact with Daam2. However, in late OLs, CK2α translocates to the cytosol where it can phosphorylate Daam2 at S704/T705. This phosphorylation interrupts the Daam2–Axin2 interaction, and the destruction complex retains β-catenin degradation. The E-mutation or CK2α overexpression weakens the Wnt signaling complex, promoting OL differentiation while negatively impacting ensheathment (*Right*). The figure was created with BioRender.com.

In this study, we present a new regulatory pathway involved in the progression of OL lineage during brain development and white matter repair. First, we demonstrated the effect of Daam2-mediated canonical Wnt signaling at specific developmental stages of the OL lineage. Second, we identified a unique posttranslational regulation that involves CK2α, which interferes with the interaction between Daam2 and Axin2 in the Wnt signaling complex through Daam2 phosphorylation. Subsequently, β-catenin is retained in the cytosol and degraded by the destruction complex, which consequently promotes OL differentiation (Fig. 7). Our white matter injury models, mimicking HIE and MS, confirm that excessive Wnt activity significantly impedes developmental and regenerative myelination by inhibiting OL differentiation. However, targeting CK2α-mediated Daam2 phosphorylation can increase the pool of available OLs and promote white matter repair, even if the process of ensheathment might be slower after injury. Overall, our findings suggest that the CK2α–Daam2 pathway plays a crucial role in the regulation of OL biology and pathology associated with Wnt signaling.

The function of Wnt signaling in OL development has been investigated in various genetic animal models (7, 12). However, due to the limitations of in vivo models, the functional complexity and stage-specific roles of Wnt and its components are not fully understood. For instance, the destruction complex proteins, GSK3β, APC, and Axin2, as well as the β-catenin transcription partner Tcf7l2, are dispensable or participate in other pathways (33–36). Recently, Tcf7l2 was proposed to serve considered as a promoter and a nuclear marker for premyelinating OLs rather than an inhibitor (37). Furthermore, the genetic approach of combining OL-specific promoters (e.g., *NG2* vs. *PLP*) with a *CreERT* system has not been used to compare early and late events in white matter development. Importantly, regulating β-catenin activation through axon 3 and 2 to 6 deletion may not recapitulate a nuclear translocation event in OL lineage, as compared to the conventional Wnt pathway in endothelial cells (38, 39). In this study, we show the stage-specific role of Wnt signaling in OL lineage progression. Expression of nuclear β-catenin levels suggests that higher endogenous Wnt activity in late stage of OLs maturation than in early OL development, which is well correlated with the Daam2 expression pattern in OL lineage progression. In addition, we prove Daam2 controls ligand-based Wnt activity in stage-specific OL development, which can be regulated by CK2α-induced Daam2 phosphorylation in vitro and in vivo. Moreover, Daam2 phosphorylation regulates different population of Wnt-related genes, including Tcf7l2, in distinct OPC/OL clusters from our scRNA-seq analysis. Our findings illustrate the dynamic mechanistic basis of canonical Wnt signaling via Daam2 in early and late OL progression.

FH2, a conserved domain in the formin protein family, can bind to F-actin and nucleate actin filaments (40). However, little is known about its function after phosphorylation modification. Although FH2 phosphorylation may not affect nucleation activity (41), FH2 phosphorylated by CK2 disrupts the interaction between formin protein FHOD3 and ubiquitin-binding scaffold protein SQSTM1 (42). This raises the possibility that S704/T705 phosphorylation at the Daam2 FH2 domain weakens the binding to unknown partners (including Axin2), which are important for Wnt signalosome complex formation (15). Axin2 is also a member of the destruction complex preventing nuclear translocation of β-catenin. Therefore, CK2α-mediated Daam2 phosphorylation could retain the activity of the destruction complex, which attenuates Wnt activity and promote OL differentiation. As a multisite regulator of Wnt, CK2α may directly control the function of the destruction complex members, such as APC. On the other hand, our recent study shows that Daam2 interacts with the E3 ligases VHL and Nedd4, which reciprocally control OL development in the spinal cord (17). It is possible that other E3 ligases can be involved in Daam2-mediated Wnt signaling, since ubiquitination can also control protein interaction and signaling (43). Here, we report the unique role of phosphorylation modification at Daam2 in OL development, yet further signaling cascade is needed for investigation.

Under normal conditions, CK2α-Daam2 interaction remains low in the cytosol compartment of OPCs and early Ols, but it increases in late OLs. Because CK2 also enhances Wnt signaling through phosphorylating Dvl and β-catenin (20), this suggests a negative feedback for Wnt when CK2α translocated to cytosol. This raises an important question about the mechanism for CK2α shuttling between the nucleus and cytosol in OLs. CK2α may form different holoenzymes with other subunits (α′ and β) in the nucleus and cytoplasm and display distinct catalytic activities and functions. Accordingly, the nuclear accumulation of β-catenin in APC-enriched mature OLs may result from cytosolic CK2α which phosphorylates β-catenin and releases it from APC and Axin2 in the destruction complex (7). On the other hand, in pathological conditions, Daam2 and Wnt signaling are up-regulated to inhibit OL differentiation (9, 17), but CK2α is restricted to the nucleus in OPCs and early OLs and unable to interact with Daam2. To overcome this challenge, we manipulated the regulatory pathway by increasing cytosolic CK2α to successfully facilitate remyelination after injury. This result highlights the translational significance of pharmaceutical reagents capable of inducing cytosolic translocation of CK2α and Daam2 phosphorylation for demyelination diseases.

Future research should further investigate the roles of CK2 in OLs. For example, CK2 blockage with cx-4945 (Silmitasertib) is a therapeutic candidate for MS for its ability to disrupt T cell development (44, 45). Additionally, inhibition of CK2 activity was found to preserve OLs from AMPA-induced toxicity (46). However, we confirm an adverse effect of CK2 inhibitors on OL differentiation. CK2α overexpression also enhances OL differentiation in vivo. Similarly, CK2β plays an essential role in NSC proliferation and OPC production (22). CK2 kinase activity is also required for gene transcription of NG2 proteoglycan, which is a marker for OPCs and required for oligodendrogenesis (47). Given that CK2β can interact with OL-specific transcription factor Olig2 in a cell-free or immortalized cell line–based assay (22), CK2α may also functionally bind to Olig2 or other transcription factors during OL development (48). Nevertheless, to fully understand the precise OL-specific targets and downstream signaling pathways of CK2α in OLs, other than Daam2, further investigation is required. Also, CK2-targeting therapies could be exploited for OL survival in the acute phase of white matter diseases and for OL differentiation in the recovery stage.

## Materials and Methods

### Materials.

All experimental materials are included in Dataset S3.

### Mice.

The Daam2 phospho-mimetic (E) mutation mouse line was generated using CRISPR/Cas9 editing and microinjection at the Baylor College of Medicine's GERM Core. Genetic mutation was confirmed by PCR using specific primers. Flag–Daam2 knock-in mice have been used in our previous study (49). All procedures were approved by the IACUC at Baylor College of Medicine and conform to the US Public Health Service Policy on Humane Care and Use of Laboratory Animals.

### Primary OPC/OL Culture.

Primary OPC culture was performed as previously described (16–17). Briefly, cortical tissues from E14.5 mouse embryos were collected and the NSCs were cultured as neurospheres for 4 d. NSCs were dissociated and transformed into OPCs in an OPC medium for 2 d. OPCs were then differentiated into OLs in an OL medium for 2 to 4 d. Prior to differentiation, OPCs were transfected with Flag-tagged Daam2 and specific plasmids for 16 h.

### scRNA-seq.

P21 mouse cortical tissues containing corpus callosum (3 animals combined per group) were dissociated with papain at 37° for 30 min. Cell solution was prepared using debris and dead cell removal kit with MS separation columns. Single-cell gene expression library was prepared according to Chromium Single Cell Gene Expression 3v3.1 kit (10× Genomics) by Single-Cell Genomic Core (SCG) at the Baylor College of Medicine. After quality control of cDNA libraries, primary data were processed with Illumina Next Generation Sequencing by Genomic & RNA Profiling Core (GARP) at the Baylor College of Medicine. The scRNA-seq data were analyzed with Cell Ranger Count v6.1.2 followed by R-studio. Cells with more than 7% mitochondrial RNA or less than 1,000 total RNA counts were excluded from data.

### In Vitro CK2 Kinase Assay.

Purified Daam2 proteins and short synthetic peptides were analyzed using ADP-Glo CK2α kinase assay. The peptides and casein (positive control) were incubated with recombinant CK2α and ATP in reaction buffer at 37° for 60 min. The levels of adenosine diphosphate (ADP) generated from phosphorylation reactions were then determined by luminescence.

### Immunofluorescence Staining.

Immunofluorescence staining was performed as previously described (16–17). Briefly, mouse tissues were fixed in 4% paraformaldehyde, dehydrated with sucrose, and embedded in optimal cutting temperature compound for sectioning. These sections were permeabilized with phosphate buffered saline containing 0.1% Tween-20 (PBST), blocked with bovine serum albumin, and incubated with primary antibodies overnight. Fluorophore-conjugated secondary antibodies and DAPI were subsequently applied. Similarly, cells on coverslips were fixed, treated with PBST, incubated with antibodies, and DAPI-stained. The prepared samples were imaged using a Zeiss Imager.M2m microscope.

### In Situ Hybridization and RNAscope.

Detection of gene transcripts in the brain tissues was performed as previous described (17). RNA probes conjugated with DIG were generated in-house for in situ hybridization. For RNAscope, RNA transcripts were visualized using the RNAscope® Multiplex Fluorescent Detection Reagents Kit v2 and RNA-protein codetection ancillary kit with the commercial probes.

### Reverse-transcription Quantitative Polymerase Chain Reaction (RT-qPCR).

RT-qPCR analysis was performed as described previously (17). Briefly, Total RNA was extracted using TRIzol. RT-qPCR was conducted in Bio-Rad real-time PCR systems using the amfiSure PCR master mix. Relative mRNA expression level was determined by the threshold cycle (Ct) value of each PCR product.

### IP and Western Blot.

Tissues or cells were homogenized in lysis buffer and incubated with antibodies and protein A/G beads overnight. The washed beads or lysates were boiled with loading buffer. Proteins were separated via sodium dodecyl-sulfate polyacrylamide gel electrophoresis and transferred to membranes. These were blotted with antibodies and visualized with ECL.

### Electron Microscopy.

Tissue preparation and processing followed our previous study (18). Regions of interest located in the corpus callosum were dissected at 1-mm^3^ blocks and postfixed with 4% PFA. The lipid content was fixed with 0.1M cacodylate solution containing 1% osmium tetroxide, 1.5% KFeCN at 4° for 1 h. Next, the tissue blocks were washed with 0.1 M cacodylate and fixed again in 2% glutaraldehyde at 4° overnight followed by washing and dehydration steps. Blocks were embedded in resin, cured, randomly numbered, and analyzed using electron microscopy.

### Neonatal Hypoxic Injury and 3-Chamber Test.

Based on the protocol from our previous study (16), P3 mouse pups and nursing mother were exposed to 10.5% oxygen for 8 d and then were returned to normoxic environment for recovery. For the 3-chamber sociability test, 2-mo-old mice were randomly number-tagged before single-blinded analysis. Mice were placed in a middle chamber which connects to chambers on the left and right side, and mice were allowed to explore the 3 chambers for 10 min in three sequential trials, respectively. The location and movement of the mice were recorded by a camera.

### Lysolecithin-Induced Demyelination.

As previously described (16, 17), mice were anesthetized and were placed on a stereotaxic device. After the skull was exposed, a hole was made at 1-mm anterior and 1-mm right to the bregma. Then, 1 μL PBS containing 2% lysolecithin and ~10^9^ U AAVs were injected into the brain at the depth of 1.5 mm. The brain containing lesion at corpus callosum was collected at 14 d post injection.

### Image Analysis, Quantification, and Statistics.

Protein signals from western blot (nonblinded), cell number counting, signal intensity, area, and Sholl analysis from immunofluorescence (single-blinded) were measured using ImageJ software with related plug-ins. For electron microscopy (single-blinded), g-ratio measurement, radius of axons with or without myelin layers were measured manually using ImageJ software. Data were analyzed, and the result plots were generated using Prism 6 software. For data comparison, student *t* test and one-way ANOVA were used when only one variable factor between groups. Two-way ANOVA with Sidak’s multiple comparison test was used for two variables among groups.

## Supplementary Material

Appendix 01 (PDF)

Dataset S01 (XLSX)

Dataset S02 (XLSX)

Dataset S03 (XLSX)

## Data Availability

The RNA-Seq dataset generated during this study are available at the NCBI Gene Expression Omnibus (GEO) website (accession: GSE236976) (26). All other study data are included in the article and/or supporting information.
