# Supplementary material for: Daam2 phosphorylation by CK2α negatively regulates Wnt activity during white matter development and injury
Source: Proc Natl Acad Sci U S A. 2023 Aug 22;120(35):e2304112120. doi: 10.1073/pnas.2304112120 (PMC10469030; doi:10.1073/pnas.2304112120)
Supplement: Supplementary file 1 — Appendix 01 (PDF) [file pnas.2304112120.sapp.pdf]

Supporting Information for

Daam2 phosphorylation by CK2α negatively regulates Wnt activity during white matter development and injury

Chih-Yen Wang, Zhongyuan Zuo, Juyeon Jo, Kyoung In Kim, Christine Madamba, Qi Ye, Sung Yun Jung, Hugo J. Bellen, and Hyun Kyoung Lee

*Corresponding author: Hyun Kyoung Lee

Email: hyunkyol@bcm.edu

This PDF file includes:

Figures S1 to S6

Other supporting materials for this manuscript include the following:

Tables S1 to S3

Fig. S1

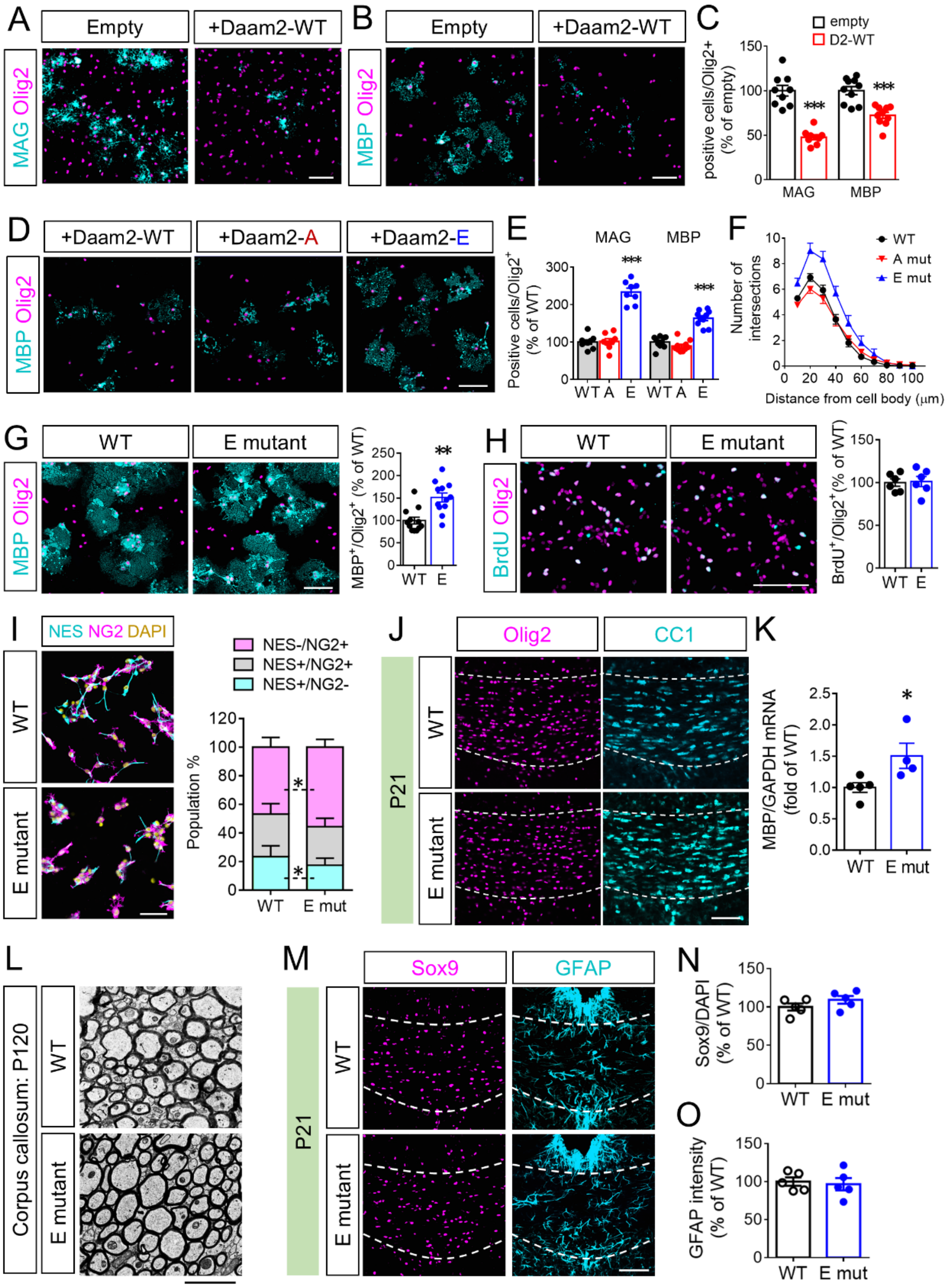

Fig. S1. Phospho-mimetic mutation of Daam2 is beneficial for OL differentiation. Primary OPCs were transfected with Flag-Daam2 and differentiated for 2 days (A) and 4 days (B, D). (C, E) In vitro differentiation was assessed by immunofluorescence for MAG and MBP. (F) The number of cell processes and branches that extend from the cell body at different distances were calculated using sholl analysis for process complexity. (G) OPCs from the E mutant were differentiated for 4 days followed by immunofluorescence for MBP. (H) WT and the E mutant

OPCs were treated with BrdU for 6 hrs before immunofluorescence to detect proliferating cells with BrdU incorporation. (I) WT and the E-mutant NSCs were differentiated into OPCs for 16 hrs followed by immunofluorescence for markers of NSCs (nestin) and OPCs (NG2). P21 corpus callosum from WT and the E mutant were analyzed by immunofluorescence (J, M) and by Q-PCR (K). (L) The myelin structure in the corpus callosum from WT and the E-mutant mice at P120 were subjected to electron microscopy. The number of Sox9+ cells was counted (N), and the immunoreactivity of GFAP was measured (O). Data from at least 3 independent experiments or animals for each group were presented as mean ± SEM. *P < 0.05, *P < 0.05, ***P < 0.001 versus empty in C, versus WT in E, K. Scale bar, 100 μm in all except L; 2 μm in L.

Fig. S2

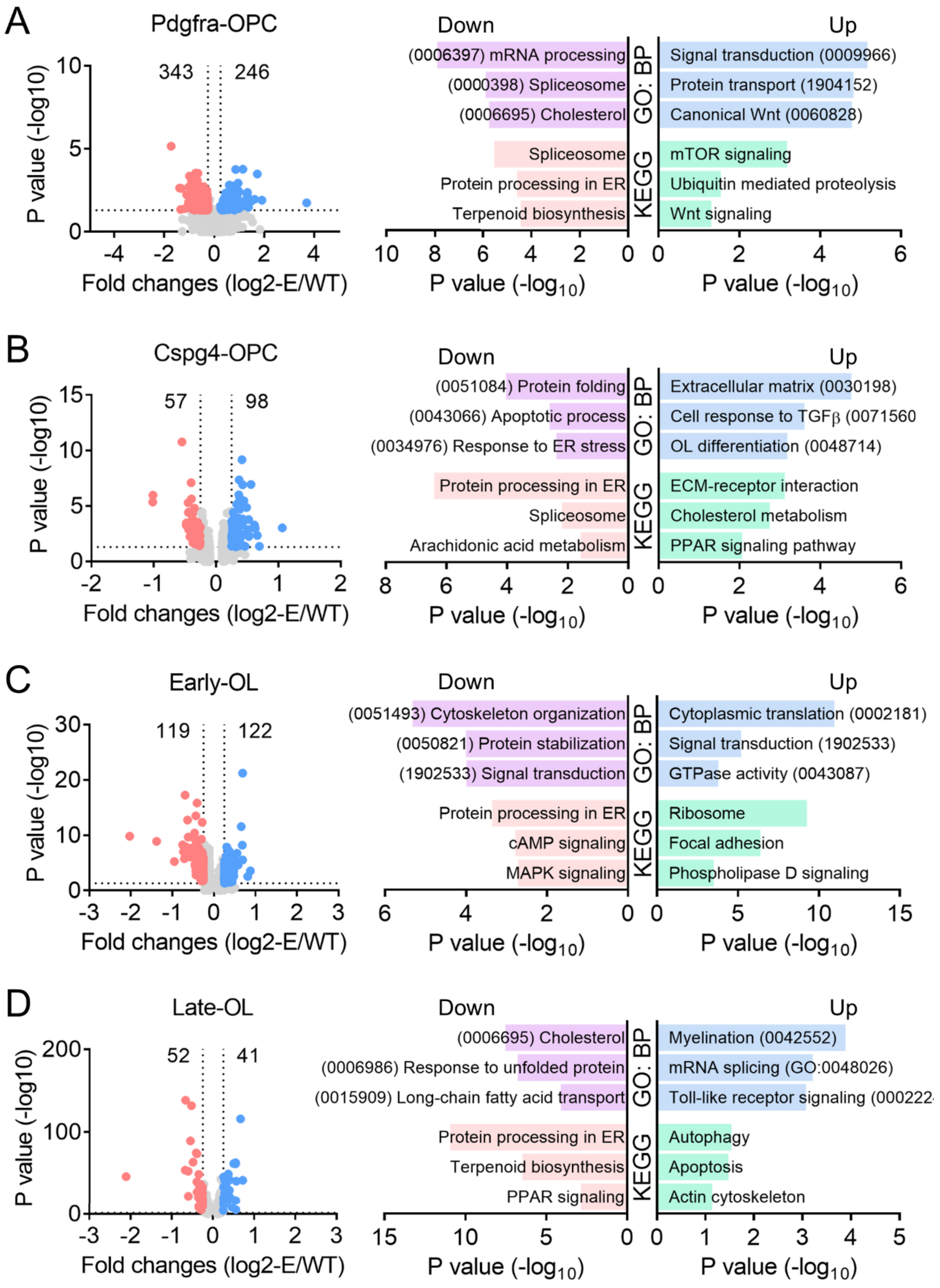

Fig. S2. Differentially expressed genes (DEGs) in 4 OPC/OL clusters between WT and the E-mutant brains. DEGs with fold change (Log_2) > 0.25 and P value < 0.05 and their numbers are shown in the volcano plots. Gene ontology and KEGG pathway analysis were performed, and the top 3 candidates related to OL biological function are listed.

Fig. S3

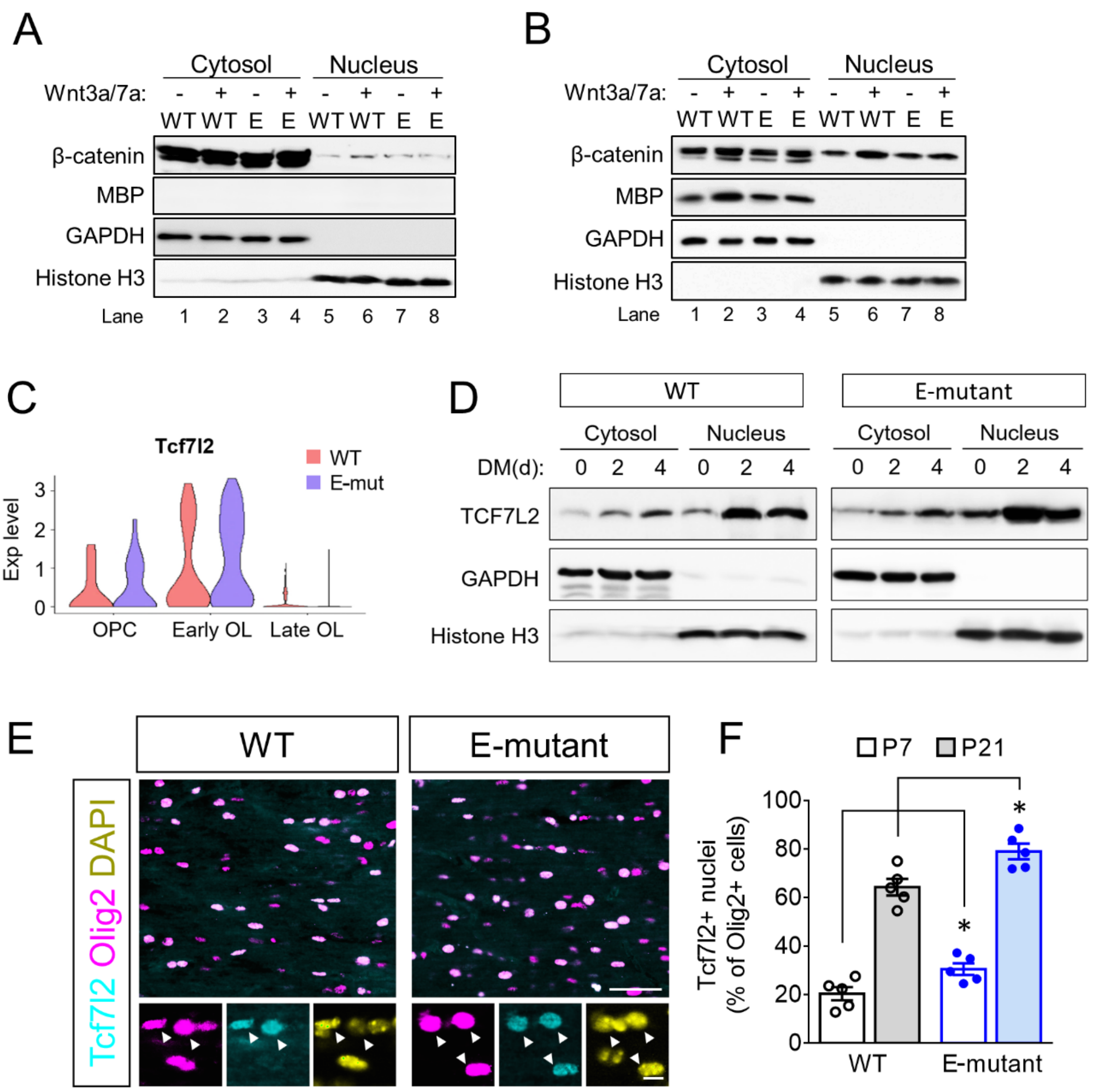

Fig. S3. Daam2-E mutation blocks ligand-based Wnt activation in OLs. (A-B) Protein levels in the cytosol and nucleus fractions of early OLs (A) and late OLs (B) were analyzed by western blot. GAPDH serves as a loading control for cytosol fractions, and Histone H3 for nucleus fractions. (C) Tcf7l2 expression in the OL clusters of the E-mutant and WT by scRNA-seq. (D) Tcf7l2 protein level was analyzed by western blot in OL culture from the E-mutant and WT mice. (E) P21 corpus callosum of the E-mutant and WT was subjected to immunofluorescence for Tcf7l2 and Olig2. (F) The number of Tcf7l2^{+}/ Olig2^{+} OLs was counted at P7 and P21. Data from at least 3 independent experiments or animals for each group were presented as mean ± SEM *P < 0.05 vs WT. Scale bar in E, 50 μm (upper panel), 10 μm (lower panel)

Fig. S4

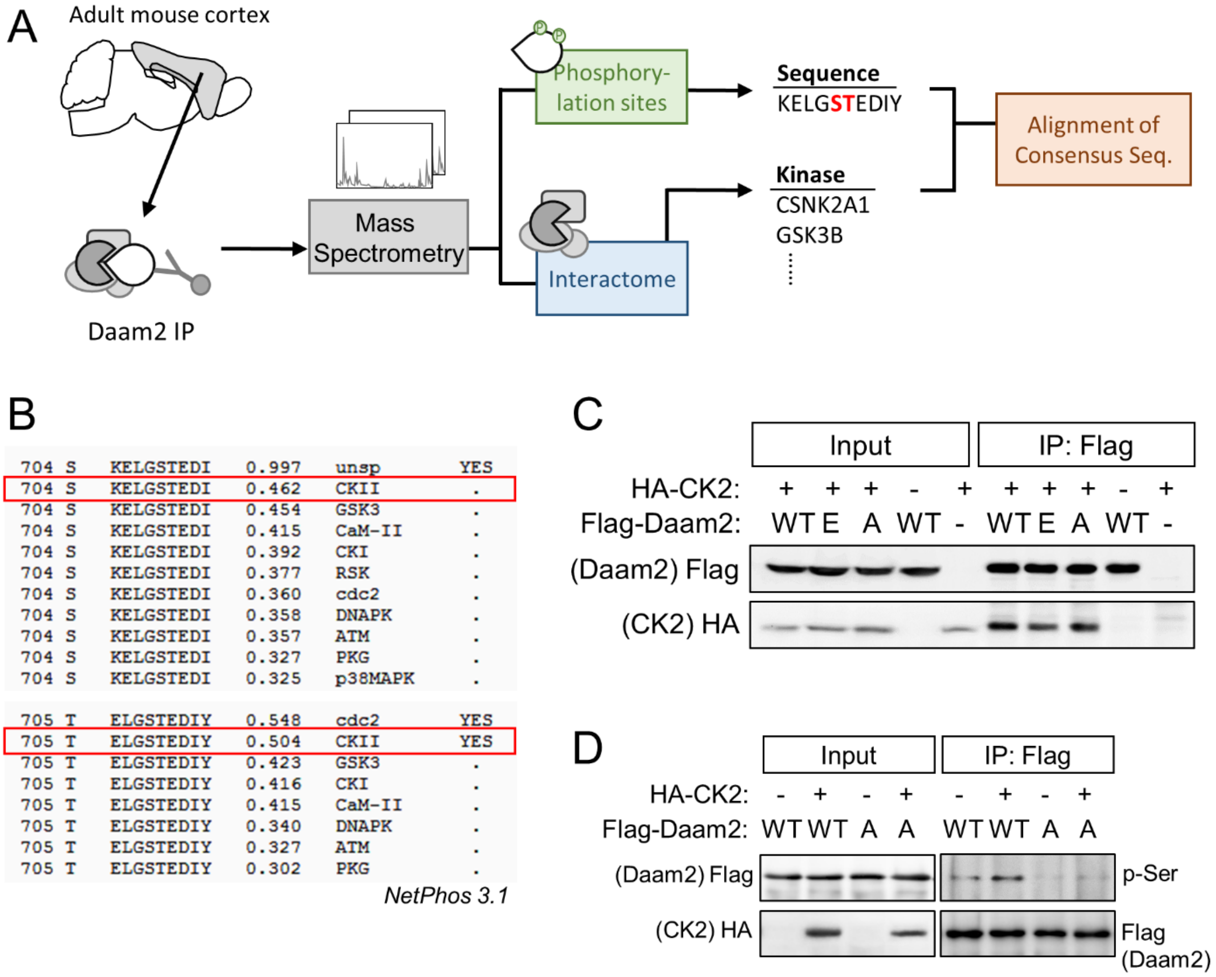

Fig. S4. CK2α is a candidate to phosphorylate Daam2 at S704/T705. (A) Experiment design for identifying candidate kinases phosphorylating Daam2. (B) The phosphorylation motif at S704/T705 was aligned in NetPhos3.1 database for kinase prediction. (C) After transfecting primary OPCs with Flag-Daam2 and HA-CK2α, HA-CK2α was co-immunoprecipitated by anti-Flag. (D) Flag-Daam2 (WT and A-mut) and HA-CK2α were transfected into OPCs followed by differentiation for 2 days. Phospho-serine (p-Ser) levels on Flag-Daam2 were analyzed by western blot after Flag immunoprecipitation.

Fig. S5

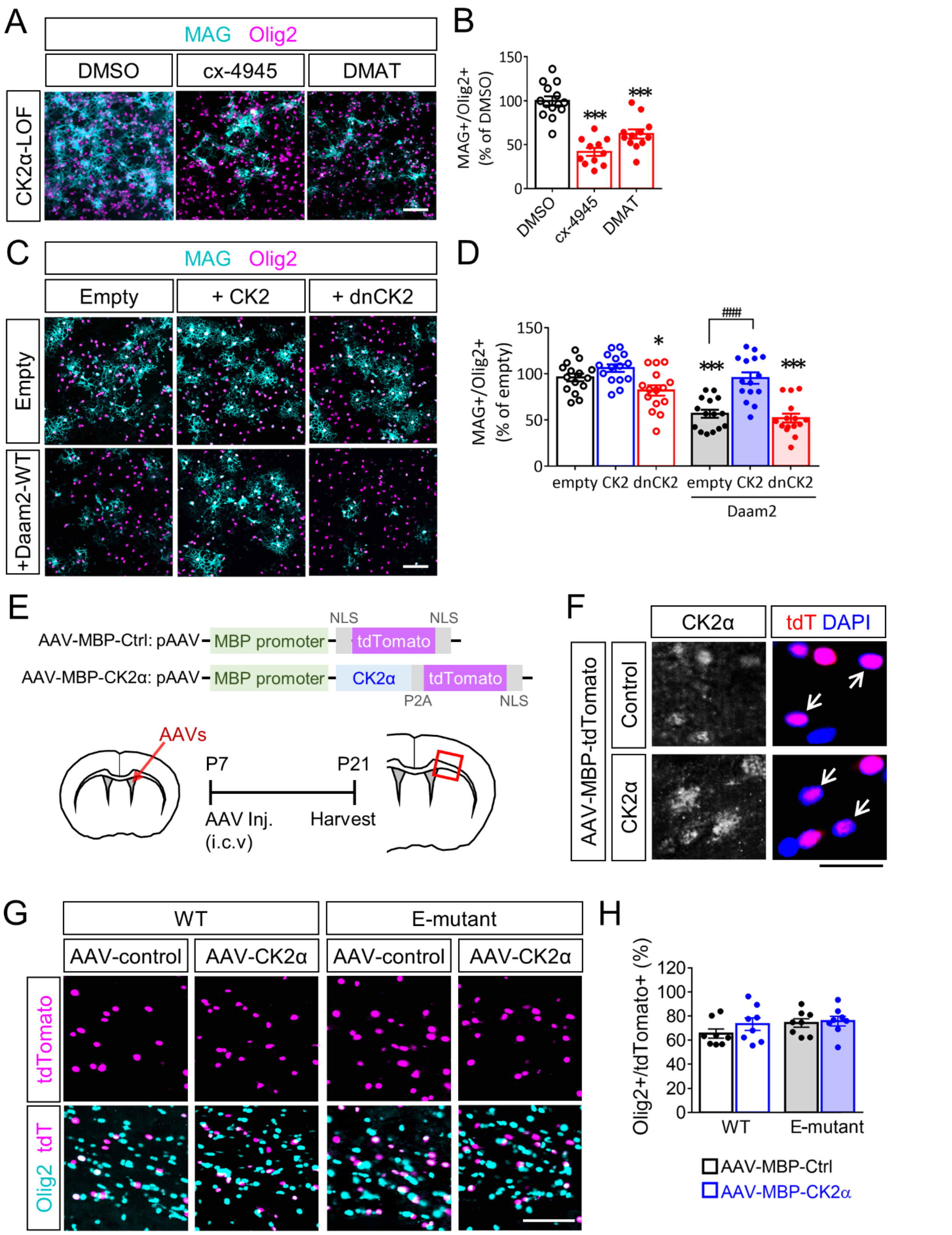

Fig. S5. CK2α kinase activity is required for OL differentiation. (A-B) Primary OPCs were treated with CK2 inhibitors and subjected to in vitro differentiation. (D) In vitro differentiation of OPCs transfected with Daam2 and CK2α (or dominant negative CK2α) were evaluated. (F) A diagram shows AAV constructs and the injection of AAVs into the brain. (G) The overexpression

of CK2α in tdtomato$^+$ cells in P21 corpus callosum injected with AAV-MBP-CK2α. (H-I) The brains injected with AAVs were assessed by immunofluorescence at P21. The number of Olig2$^+$ and tdtomato$^+$ cells in the corpus callosum were counted. Data from at least 3 independent experiments or animals for each group were presented as mean ± SEM. *$P < 0.05$, ***$P < 0.001$ versus DMSO in B, versus empty in E; $^{###}P < 0.001$ versus Daam2 in E. Scale bar, 100 μm in A, C; 20 μm in F; 100 μm in G.

Fig. S6

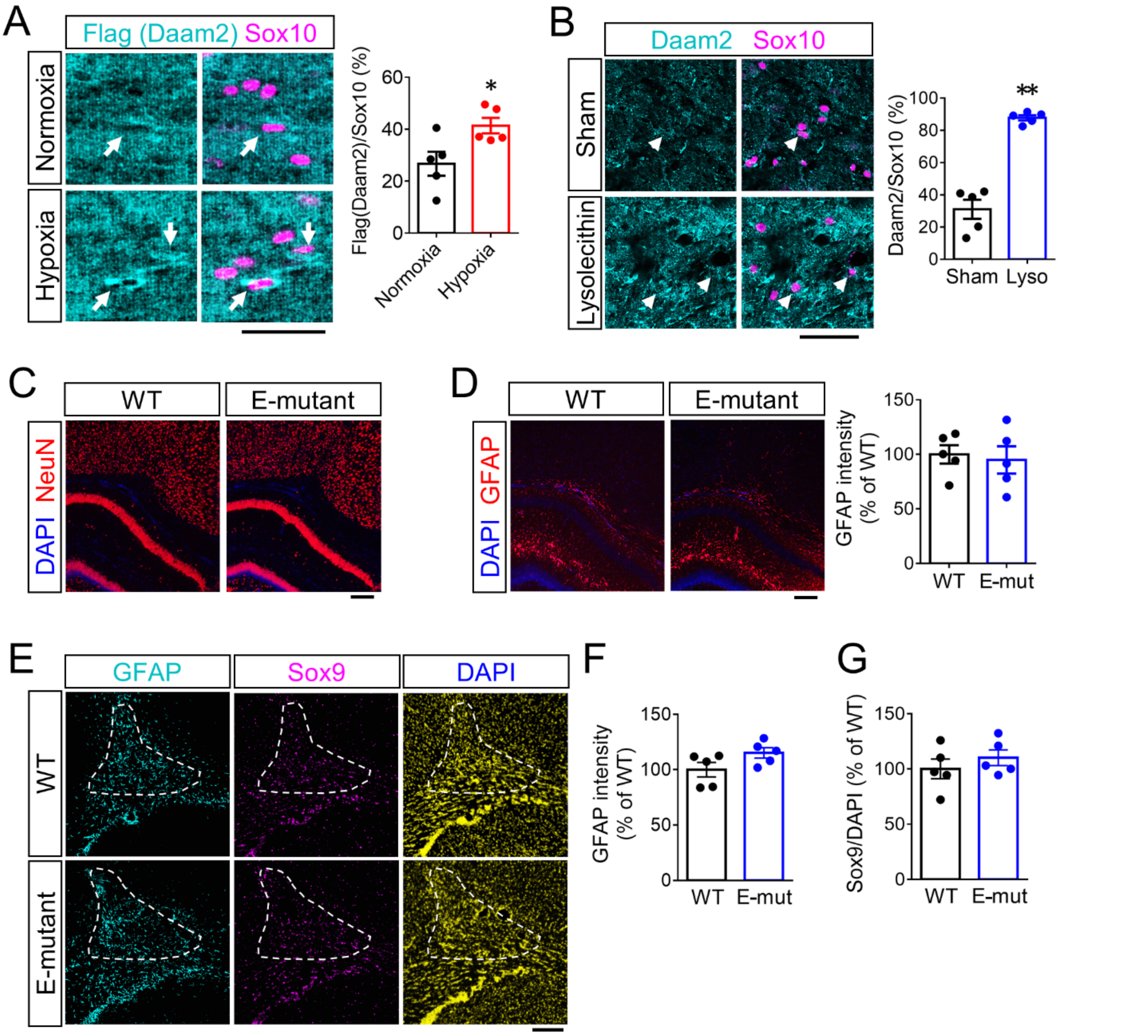

Fig. S6. Daam2 is upregulated after white matter injury. P11 Flag-Daam2 knock-in brains after postnatal hypoxic injury (A) and the adult brains 14 days after lysolecithin injection (B) were subjected to immunofluorescence. Intensity of Daam2 (or Flag) in Sox10$^+$ cells in the corpus callosum was measured. (C-D) NeuN$^+$ neurons and GFAP$^+$ astrocytes were assessed in P18 brains after postnatal hypoxic injury. (E-G) GFAP$^+$ and Sox9$^+$ astrocytes were assessed 14 days after lysolecithin injection. Data from at least 5 independent experiments or animals for each group were presented as mean ± SEM. *P < 0.05, **P < 0.01 versus normoxia in A, versus sham in B. Scale bar, 50 μm in A, 100 μm in B, 200 μm in C-E.

Dataset S1 (separate file). Comparison of OL-related gene expression in the early and late OL clusters from scRNA-seq. The average fold change (Avg_Log2) of gene expression in the E-mutant versus WT are listed (Pink, significantly upregulated; Blue, significantly downregulated; $P < 0.05$). The percentage of cells (Pct) in the E-mutant and WT that were positively expressing the gene are also shown.

Dataset S2 (separate file). Wnt-related genes in OL clusters from scRNA-seq. The percentages of the altered genes (%) in each OL cluster that are associated with Wnt signaling were calculated. The numbers of datasets (Wnt #) in which the gene is present, were counted. The Wnt datasets used in this study are provided.

Dataset S3 (separate file). Materials. The information of antibodies, chemicals, nucleotides, animals, and software that have been used in this study are listed.
